# TSH receptor antibodies have predictive value for breast cancer – retrospective analysis

**DOI:** 10.1186/1756-6614-6-8

**Published:** 2013-05-16

**Authors:** Paweł Szychta, Wojciech Szychta, Adam Gesing, Andrzej Lewiński, Małgorzata Karbownik-Lewińska

**Affiliations:** 1 Department of Oncological Endocrinology, Medical University of Lodz, 7/9 Zeligowski St., 90-752, Lodz, Poland; 2 Department of Endocrinology and Metabolic Diseases, Medical University of Lodz, Lodz, Poland; 3 Department of Endocrinology and Metabolic Diseases, Polish Mother’s Memorial Hospital, Research Institute, Lodz, Poland; 4 Current address: Department of Oncological Surgery and Breast Diseases, Polish Mother’s Memorial Hospital, Research Institute, Lodz, Poland; 5 Current address: 1st Department of Cardiology, Medical University of Warsaw, Warsaw, Poland

**Keywords:** Breast cancer, Thyroid, Autoimmune disease, Graves’ disease, TSH receptor antibody, TSH, Thyroglobulin antibody, Thyroperoxidase antibody

## Abstract

**Background:**

Associations between breast cancer and thyroid disorders are reported in numerous studies. Relationships between thyroperoxidase antibodies (TPOAb), thyroglobulin antibodies (TgAb) and breast cancer have been previously demonstrated. However, no analysis has been performed concerning an association between thyrotropin (TSH) receptor antibodies (TSHRAb) and breast cancer. The aim of the study was to evaluate the prevalence of breast cancer or benign breast tumors in patients with Graves’ disease and to analyze a possible relationship between Graves’ disease and these two groups of breast diseases with emphasis to epidemiology and laboratory findings.

**Patients and methods:**

Clinical and laboratory details of 2003 women hospitalized for endocrine disorders were retrospectively analyzed, using an unpaired Student’s *t*-test, logistic regression analysis, *χ*^2^ test of independence or the two-sided ratio comparison test.

**Results:**

The coexistence of Graves’ disease and breast cancer was statistically significant. We observed TSHRAb and TgAb more frequently in patients with breast cancer. We found that TSHRAb is the only variable possessing predictive value for breast cancer.

**Conclusions:**

The strong relationship between Graves’ disease and breast cancer is proposed. We suggest that TSHRAb could be described as a positive determinant of breast cancer. The present data call attention to the usefulness of screening for breast cancer in long-term follow-up of patients with autoimmune thyroid disorders, especially of those with Graves’ disease. Similarly, screening for autoimmune thyroid disorders should be performed in patients with nodular breast disease. Additionally, the article draws ideas for further research in order to develop targeted treatment for more successful outcome in patients with breast cancer.

## Introduction

Breast cancer is a hormone dependent malignancy. Thyroid hormone receptors affect both the normal breast cell differentiation and breast cancer cell proliferation, with effects of thyroid hormones similar to those caused by estrogens [1,2]. Relationship between thyroid diseases, such as nodular hyperplasia, hyperthyroidism and thyroid cancer, with breast cancer was demonstrated in several studies [3-6]. However, ambiguous results concerning the above association have been recently summarized [7]. In contrast, hypothyroidism due to Hashimoto's thyroiditis was documented as a protective factor against breast cancer [8-10], but also this observation was not confirmed in other sources [11].

Graves' disease, one of the thyroid autoimmune diseases, is characterized – in its typical form – by hyperthyroidism with laboratory results of decreased thyrotropin (TSH) level, increased free thyroxine (FT_4_) and/or free triiodothyronine (FT_3_) levels, detectable TSH receptor (TSHR) stimulating antibodies (TSHRAb), usually positive thyroid peroxidase antibodies (TPOAb) and thyroglobulin antibodies (TgAb) [12]. An exclusive diagnostic feature of Graves' disease is the presence of TSHRAb. The ligand for TSHRAb, i.e. TSHR, is also present in breast cancer tissue [13].

Only limited aspects of potential association between Graves’ disease and breast cancer have been postulated [14,15], whereas the exact mechanism has not been identified [16]. Genetic, environmental and molecular pathways of both female predominant diseases have been described, and integrated analysis of the above entities provides opportunity to identify the potential relevant common etiological mechanisms [17]. The potential relationship between antithyroid autoantibodies and breast cancer has not been clearly documented, as the elevated serum levels of TPOAb and TgAb in patients with breast cancer, detected in some studies [18-21], have not been confirmed elsewhere [22,23]. Moreover, no conclusive research has been undertaken concerning significance of TSHRAb in patients with breast cancer [24].

The aim of the study was to evaluate the prevalence of breast cancer or benign breast tumors in patients with Graves’ disease and to analyze a possible relationship between Graves’ disease and these two groups of breast diseases with emphasis to epidemiology and laboratory findings.

## Patients and methods

Retrospective clinical details of 2003 women, who were hospitalized for endocrine disorders in the Department of Endocrinology and Metabolic Diseases at the Polish Mother’s Memorial Hospital – Research Institute in Lodz within a 3-year period between 2002 and 2005, were retrieved from the hospital records following the internal audit approval. Inclusion criteria were female adults. Exclusion criteria were all oncological conditions other than breast neoplasia and thyroid disorders other than Graves’ disease, such as nodular goiter, thyroid cancer, autoimmune thyroiditis (AIT), etc.

After exclusion, 1686 women aged 36.48 ± 15.95 years were enrolled to the study (Table 1). Two studied groups consisted of 47 patients with benign breast tumors (BBT), aged 46.27 ± 14.18 years and 9 patients with breast cancer (BC), aged 54.55 ± 9.60 years. Therefore, 1630 women hospitalized for several non-oncological diseases and without thyroid diseases other than Graves’ disease (polycystic ovary syndrome, primary infertility, osteopenia, osteoporosis, obesity, dwarfism, anorexia, atherosclerosis, cardiomyopathy, dyslipidemia, hirsutism, hypertension, ischemic heart disease, gastric ulcer, metabolic syndrome or diabetes of any type) were considered as a control group (C) with an average age of 36.10 ± 15.89 years.

**Table 1:** Clinical characteristics of patients with breast cancer, benign breast tumors, Graves’ disease and in controls

**Parameter**	**Clinical characteristics**				**Statistical analysis**		
	**BC**	**BBT**	**C**	**All patients**	**p (BC vs. BBT)**	**p (BC vs. C)**	**p (BBT vs. C)**
**Patients** – No (%)	9 (0.53%)	47 (2.78%)	1630 (96.6%)	1686 (100%)	ns	<0.0001	<0.0001
**Age** – yr (mean ± SD)	54.55 ± 9.60	46.27 ± 14.18	36.10 ± 15.89	36.48 ± 15.95	ns	0.0005	<0.0001
**Graves’ disease –** No (%)	3 (33.3%)	6 (12.7%)	111 (6.8%)	120 (7.1%)	ns	0.0025	ns

Clinical characteristics of patients with breast cancer (BC), patients with benign breast tumors (BBT) and controls (C) and the occurrence of Graves’ disease. Statistical evaluation was done by an unpaired Student’s *t*-test (for age) or by the two-sided ratio comparison test (for no. of patients and for Graves’ disease occurrence); ns, non-significant.

Because of the relatively high age range of women with breast cancer and to analyze the cause-effect relation, age-matched groups of patients and healthy women could not be investigated in all stages of the analysis. Therefore, the predictors of breast cancer were analyzed in the age-matched group of 499 patients, including 9 patients with BC aged 54.55 ± 9.60 years and 490 control individuals (C) aged 53.86 ± 8.88 years (p > 0.05).

The following parameters were recorded with the use of the data analysis program, designed by one of co-authors of the study (P.S.): age and laboratory parameters, i.e. TSH, FT_4_, FT_3_, TSHRAb, TgAb and TPOAb. The following diagnoses were considered: Graves’ disease, benign breast tumors (BBT) and breast cancer (BC). The normal ranges of the laboratory tests in our hospital are: TSH (0.27–4.2 mIU/L), FT_4_ (0.93–1.7 ng/dL), FT_3_ (1.8–4.6 pg/mL), TPOAb (<35 IU/mL), TgAb (<115 IU/mL) and TSHRAb (<1.8 IU/mL).

The data were statistically analyzed using an unpaired Student’s *t*-test – for continuous variables. The two-sided ratio comparison test was used to evaluate the frequency of events. Univariate logistic regression analysis, followed by multivariate logistic regression analysis, was used to determine which continuous variable might have predicted breast cancer or benign breast tumors. The *χ*2 test of independence was used to determine, which dichotomized variable might have predicted breast cancer or benign breast tumors. The results are presented as mean ± standard deviation (SD). Statistical significance was determined at the level of p < 0.05.

## Results

Statistically significant differences were found in age distribution between patients with breast cancer (BC) and control group (C), as well as between women with benign breast tumors (BBT) and control group (C) (Table 1). However, there were no statistical differences in age distribution between patients with benign breast tumors (BBT) and patients with breast cancer (BC).

Graves’ disease was diagnosed on the basis of clinical findings and the presence of TSHRAb in 120 (7.11%) female patients aged 41.65 ± 15.19 years. Graves’ disease coexisted more frequently with breast cancer (p = 0.0021), but not with benign breast tumors (p > 0.05) (Table 2).

**Table 2:** Graves’ disease as the dichotomized determinant of breast cancer or benign breast tumors

**Dichotomized determinant**	**Association with BC (n = 9)**		**Association with BBT (n = 47)**	
	***χ***^**2**^	**p**	***χ***^**2**^	**p**
**Graves’ disease (n = 120)**	9.41	0.0021	2.33	ns

*χ*^2^ test of independence analysis of the dichotomized determinant (i.e. Graves’ disease occurrence) of breast cancer (BC) or of benign breast tumors (BBT), performed in all patients; ns, non-significant in all patients.

**Figure 1:**
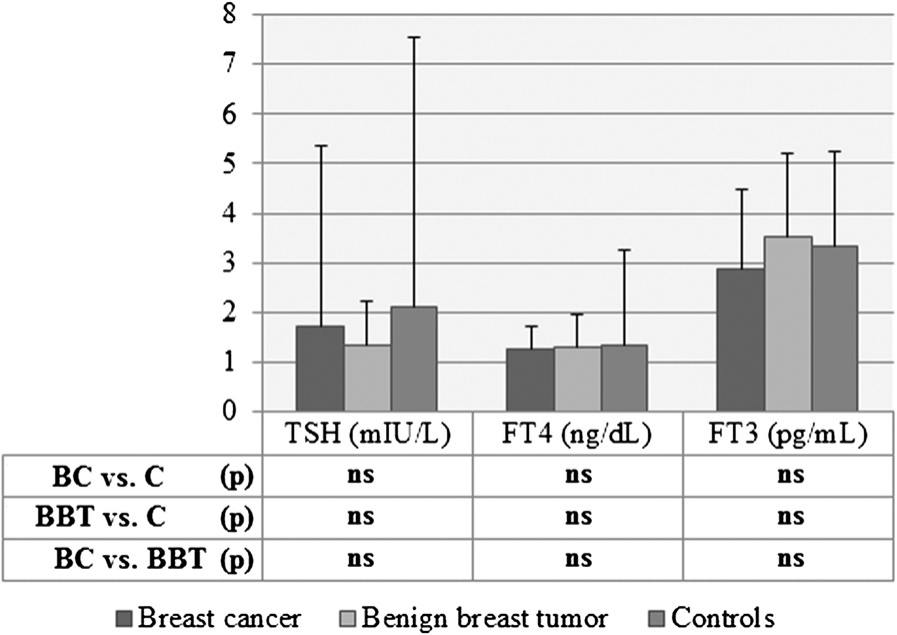
**Levels of hormones in patients with breast cancer, with benign breast tumors and in controls.** Levels of hormones in: patients with breast cancer (BC), patients with benign breast tumors (BBT) or controls (C), expressed as mean ± SD; Statistical analysis with Student’s unpaired *t*-test; ns, non-significant.

In breast cancer patients, the mean values for TSH were 1.724 ± 3.629 mU/L and they did not differ significantly from those in controls (2.12 ± 5.41 mU/L) and from those in patients with benign breast tumors (1.35 ± 0.89 mU/L) (Figure 1). In turn, FT_4_ was 1.283 ± 0.427 ng/dL in patients with breast cancer and did not differ significantly from levels found either in controls (1.34 ± 1.93 ng/dL) or in women with benign breast tumors (1.30 ± 0.67 ng/dL). In patients with breast cancer, FT_3_ was 2.882 ± 1.617 pg/mL and was similar to controls (3.33 ± 1.91 pg/mL) and to patients with benign breast tumors (3.54 ± 1.65 pg/mL).

**Figure 2:**
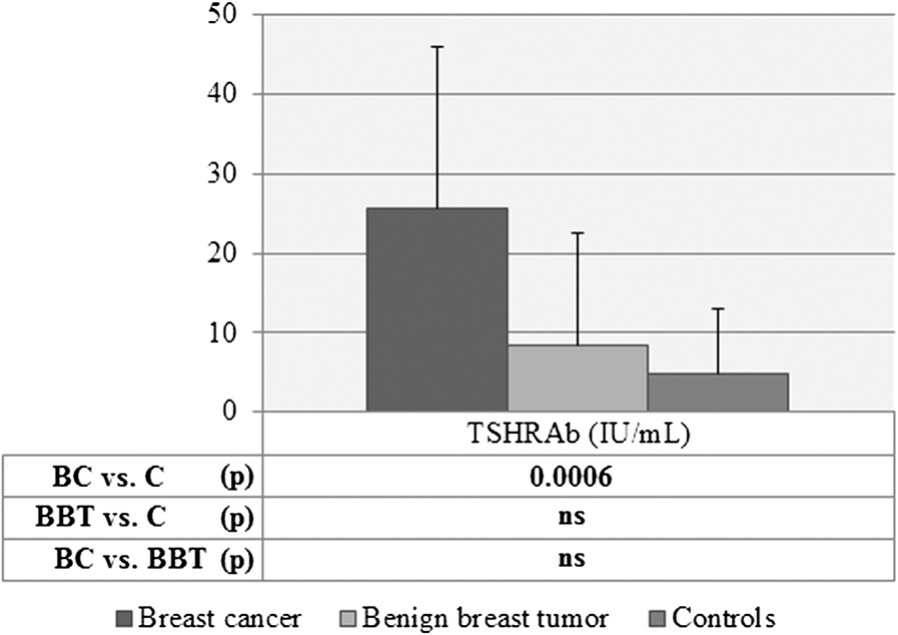
**Levels of TSHRAb in patients with breast cancer, with benign breast tumors and in controls.** Levels of TSHRAb in: patients with breast cancer (BC), patients with benign breast tumors (BBT) or controls (C), expressed as mean ± SD; Statistical analysis with Student’s unpaired *t*-test; ns, non-significant.

**Figure 3:**
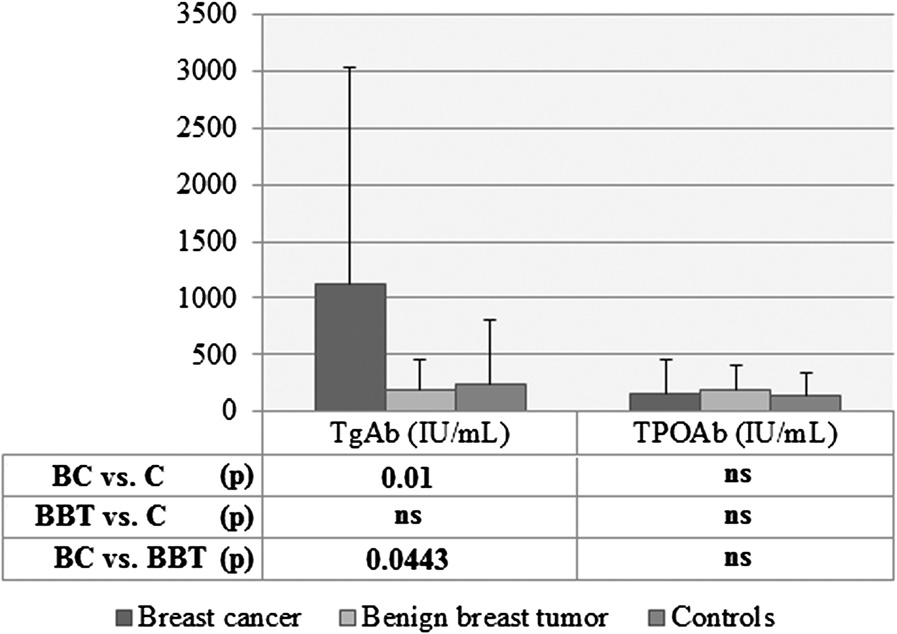
**TgAb and TPOAb levels in patients with breast cancer, benign breast tumors and in controls.** Levels of TgAb and TPOAb in: patients with breast cancer (BC), patients with benign breast tumors (BBT) or controls (C), expressed as mean ± SD; Statistical analysis with Student’s unpaired *t*-test; ns, non-significant.

When levels of thyroid antibodies were compared among three groups of patients, the following differences were found. The mean values of serum TSHRAb were 25.65 ± 20.29 IU/ml in breast cancer patients, they were significantly lower in controls (4.83 ± 8.19 IU/ml, p = 0.0006), and were lower in patients with benign breast tumors (8.52 ± 14.04 IU/ml), but the differences between BC and BBT or between BBT and controls did not reach statistical significance (Figure 2). Serum concentrations of TgAb in patients with breast cancer were 1120.8 ± 1914.9 IU/ml and were considerably higher than in controls (241.4 ± 565.0 IU/ml, p = 0.0100) and markedly higher than in patients with benign breast tumors (191.7 ± 267.1 IU/ml, p = 0.0443) (Figure 3). In turn, TPOAb in patients with breast cancer were at level of 157.8 ± 294.8 IU/ml and were similar to control group (135.7 ± 206.0 IU/ml, p > 0.05) and to benign breast tumors (178.9 ± 221.9, p > 0.05).

Several parameters, such as hormones and antibodies concentrations were submitted to a univariate and a multivariate logistic regression model. The purpose of the model was to determine which of those continuous variables might predict breast cancer or benign breast tumors either in the entire group of female patients or in age-matched groups. No predictive value for any of the examined hormones and for TPOAb was documented at univariate regression analysis in the entire group of patients (Table 3). In opposite, breast cancer predictive value for TSHRAb (OR = 1.10, 95% CI = 1.01-1.20, p = 0.0222) and TgAb (OR = 1.00, 95% CI = 1.00-1.01, p = 0.0377) was found at univariate regression analysis in the entire group of patients. Thus, TSHRAb and TgAb could be theoretically considered as positive risk factors for breast cancer. However, both above determinants lost their predictive value at multivariate analysis. In turn, meticulous logistic regression analysis, based on the age-matched groups of patients with breast cancer and controls, revealed that TSHRAb is the only variable possessing predictive value for breast cancer (OR = 1.09, 95% CI = 1.00-1.20, p = 0.0368) (Table 4). No other predictors of breast cancer or benign breast tumors were documented in our series.

**Table 3:** Hormone and autoantibody concentrations as determinants of breast cancer or benign breast tumors

**Parameter**	**Association with BC (n = 9)**						**Association with BBT (n = 47)**		
	**Univariate logistic regression**			**Multivariate logistic regression**			**Univariate logistic regression**		
	**OR**	**95% CI**	**p**	**OR**	**95% CI**	**p**	**OR**	**95% CI**	**p**
**TSH (mIU/L)**	0.97	0.75-1.26	ns	-			1.20	0.92-1.55	ns
**FT**_**4 **_**(ng/dL)**	0.96	0.42-2.21	ns				1.01	0.78-1.31	ns
**FT**_**3 **_**(pg/mL)**	0.48	0.71-1.82	ns				0.95	0.81-1.11	ns
**TSHRAb (IU/mL)**	1.10	1.01-1.20	0.0222	0.92	0.66-1.28	ns	0.97	0.91-1.03	ns
**TgAb (IU/mL)**	1.00	1.00-1.01	0.0377	0.99	0.99-1.00	ns	1.00	0.99-1.00	ns
**TPOAb (IU/mL)**	1.00	0.99-1.01	ns	-			1.00	0.99-1.00	ns

Univariate and multivariate logistic regression analysis of the univariate determinants (such as hormone and autoantibody concentrations) of breast cancer (BC) or breast benign tumor (BBT), performed in all patients (n = 1686); OR, odds ratio; CI, confidence interval; ns, non-significant.

**Table 4:** Hormone and autoantibody concentrations as breast cancer or benign breast tumors determinants, in age-matched groups

**Parameter**	**Association with BC (n = 9)**		
	**OR**	**95% CI**	**p**
**TSH (mIU/L)**	0.96	0.77-1.21	ns
**FT**_**4 **_**(ng/dL)**	0.92	0.32-2.67	ns
**FT**_**3 **_**(pg/mL)**	0.69	0.29-1.62	ns
**TSHRAb (IU/mL)**	1.09	1.00-1.20	0.0368
**TgAb (IU/mL)**	1.00	0.99-1.00	ns
**TPOAb (IU/mL)**	0.99	0.99-1.00	ns

Univariate logistic regression analysis of the univariate determinants (such as hormone and autoantibody concentrations) of breast cancer (BC) or breast benign tumor (BBT), performed in the age-matched groups of patients (n = 499); OR, odds ratio; CI, confidence interval; ns, non-significant.

**Figure 4:**
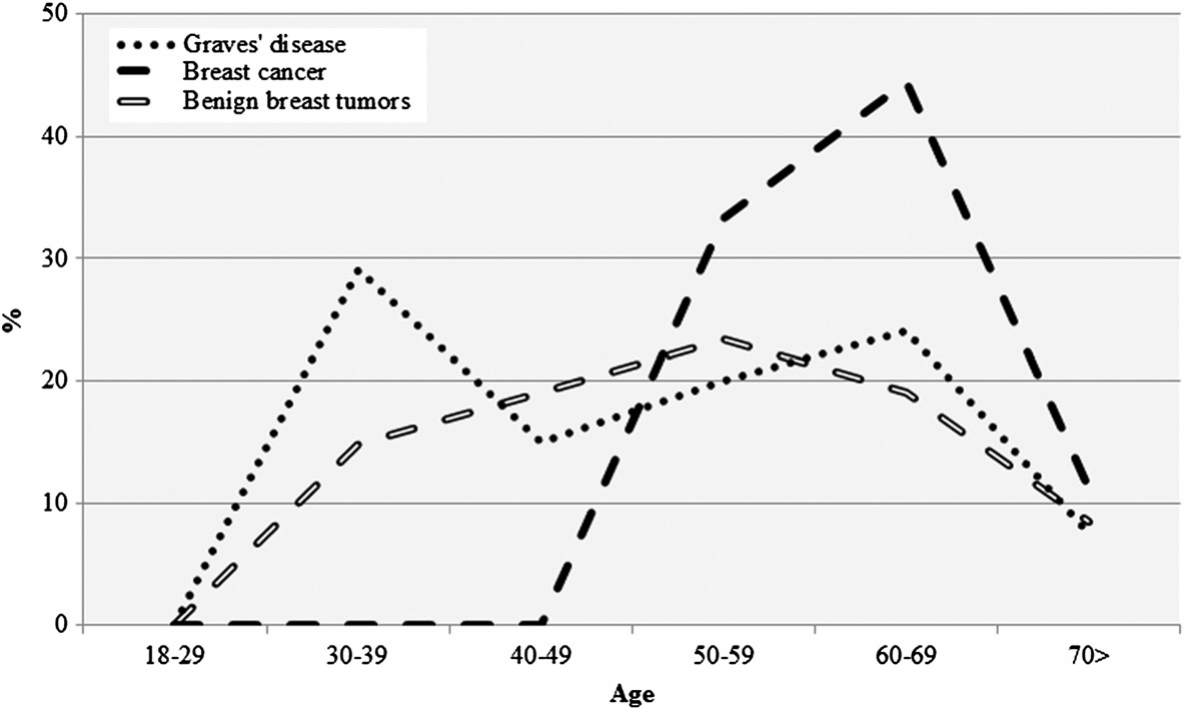
**Trends in occurrence of breast cancer, benign breast tumors and Graves’ disease.** Distribution of breast cancer (BC) or benign breast tumors (BBT) in relation to age intervals and in comparison to Graves’ disease, expressed as linear trends.

Distribution of breast tumors in relation to age intervals and in comparison to Graves’ disease is presented in Figure 4. A shift between diagnosis of Graves’ disease and breast cancer was observed. Peak incidence of Graves’ disease was seen in patients at age of about 35 years and the lowest frequency was observed at age of about 45 years, whereas peak level of breast cancer diagnosis were noted at about 65 years, and the lowest frequency at about 75 years. In contrast, we observed no similar distribution trends in relation to age intervals between benign breast tumors and breast cancers or between benign breast tumors and Graves’ disease.

## Discussion

Mammary gland is derived from iodide-concentrating ectoderm [17]. Breast functions are similar to the thyroid gland in relation to the absorption capacity of iodide for use as a milk ingredient during lactation [4,25]. Altered thyroid hormonal function due to abnormal iodine uptake has an impact on incidence of iodine deficiency disorders (IDD) [25], autoimmune diseases and could possibly affect cancer development. Increased intake of iodine is considered as a protective factor against the occurrence of breast cancer [26]. In conformity, low levels of iodine were described in the breast cancer tissues as compared to normal breast tissue or benign breast tumors [27].

Uptake of serum iodide into the breast alveolar and ductular cells happens in the mechanism of active transport via the glycoprotein – Na+/I-symporter (NIS) [28]. The expression of NIS occurs in 80% to 90% of breast cancer cases and, thus, symporter could be potentially used in the radioisotope breast imaging with ^125^I (alternatively ^99^mTc) and in the breast cancer treatment with ^131^I (alternatively ^188^Re) [13], following administration of stimulants enhancing NIS expression [29,30].

Assessment of hormonal thyroid function in breast cancer patients was assessed in our study with measurement of TSH, FT_4_ and FT_3_ levels and we found no differences among breast cancer patients, women with benign breast tumors and female controls. However, limited short-term hormone observations cannot be interpreted in relation to the long-term cancer development because of the changeable thyroid activity affected by several extrinsic and intrinsic factors. In turn, concerning the impact of autoimmune diseases on breast cancer incidence, we confirmed the high prevalence of breast cancer in patients with Graves' disease (Table 2).

Thyroid antibodies in the autoimmune thyroid disorders could interact with the receptors on breast tumors and thus they were previously designated as precursors for the coincidence of mammary and thyroid disorders [31]. Autoantigens, TPO in the thyroid and lactoperoxidases in the breast, are required for organification of iodine to produce iodoproteins [32], as they catalyze H_2_O_2_ production to oxidize I^-^[33]. Previously reported potential association between TPOAb or TgAb and breast cancer was arguable and the results were summarized in Table 5[4,18,34-36]. The presence of TPOAb was previously described as a prognostic index of a more favorable outcome in patients with breast cancer, with similar importance to the tumor size and to the axillary nodal status [37]. In our study, TPOAb levels were similar among the three analyzed groups. In turn, TgAb levels were higher in our series in patients with breast cancer comparing to controls and to patients with benign breast tumors. However, the ligand for TgAb, thyroglobulin (Tg), has antigenicity affected by the iodine content within the protein [18,38], and thus TgAb are present non-specifically in different diseases coexisting with the altered iodine intake [39]. More importantly, TgAb can be also found in patients with chronic disorders not involving thyroid [40] or even in healthy patients without any thyroid disease [41]. Thus, clinical impact of the elevated concentrations of TgAb is unreliable, as they were detected non-specifically to thyroid diseases or breast cancer.

**Table 5:** Summarized previous reports concerning relationship between thyroid diseases and breast cancer or benign breast tumors

**Parameter**	**[Previously reported data] (n = number of patients enrolled to study)**														
	[3]**(n = 150)**			[18]**(n = 115)**			[34]**(n = 61)**			[35]**(n = 175)**			[36]**(n = 48)**		
	**BC**	**C**	**p**	**BC**	**C**	**p**	**BC**	**C**	**p**	**BC**	**C**	**p**	**BC**	**C**	**p**
**n**	150	100	-	66	49	-	36	100	-	100	75	-	26	22	-
**Age-yr means ± SD (min-max)**	63 (38–80)	age-matched	-	63.5 ± 11.8	68.4 ± 12.8	ns	52.8 ± 10.2	age-matched	-	63 (38–80)	-	-	(30–85)	age-matched	-
**TSH**	3.12 ± 1.40	1.46 ± 0.82	ns	1.77 (0.15-47.9)	1.52 (0.45-14.56)	ns	1.9 ± 0.7	1.8 ± 1.4	ns	4.12 ± 1.40	1.39 ± 0.79	**0.030**	1.36 ± 0.63	2.41 ± 0.35	<0.05
***units***	*μIU/ml*			*mIU/dl*			*μIU/ml*			*μIU/ml*			*μU/ml*		
**FT**_**4**_	2.64 ± 0.91	1.42 ± 0.31	ns	15.70 (7.38-22.63)	15.19 (10.5-21.5)	ns	9.76 ± 2.73	9.9 ± 2.4	ns	2.93 ± 0.57	1.39 ± 0.21	**0.030**	1.40 ± 1.64	1.10 ± 0.83	<0.05
***units***	*ng/dl*			*pmol/dl*			*pg/ml*			*ng/dl*			*ng/dl*		
**FT**_**3**_	8.47 ± 0.75	4.48 ± 0.75	ns	-			3.58 ± 0.7	3.2 ± 0.6	ns	7.25 ± 0. 75	3.42 ± 0.91	**0.030**	3.56 ± 3.14	2.87 ± 3.12	<0.001
***units***	*pmol/l*						*pg/ml*			*pmol/l*			*pmol/ml*		
**TgAb**	140.92 ± 21.52	27.75 ± 7.60	ns	35.80 (26.1-6000.0)	27.70 (18.5-298.0)	**<0.001**	12/36	12/100	**p < 0.01**	-			-		
***units***	*IU/ml*			*kIU/dl*			*Patients’ ratio with positive TgAb*								
**TPOAb**	105.82 ± 21.46	23.08 ± 4.16	**0.030**	6.10 (6.1-871.0)	6.10 (6.1-1621.6)	ns	12/36	8/100	**p < 0.01**	104.57 ± 19.39-	24.81 ± 5.16	**0.030**	-		
***units***	*IU/ml*			*kIU/dl*			*Patients’ ratio with positive TPOAb*			*IU/ml*					
**Diagnosis**	**BC**	**C**	**p**	**BC**	**C**	**p**	**-**								
**Graves’ disease**	-			0/66	0/49	-									
**AITD**	57/150	17/100	**0.001**	16/66	8/49	-									

Summary of the previously reported endocrine and immunological laboratory status in patients with breast cancer (BC) and controls (C); ns, non-significant.

Relationship between incidence of benign breast tumors and thyroid diseases with the increased levels of TgAb and TPOAb in serum has been also proposed [42]. Previous report documented the increased level of TPOAb in 28% of women with benign fibrocystic mastopathies and 80% had thyroid hypertrophy [21,43]. In contrary, we did not observe any association between Graves’ disease, hormones or antibodies concentrations and occurrence of benign breast tumors.

Interaction between thyroid and breast cancer can occur in the mechanism involving TSHR, common in the adipose breast tissue [35]. Under physiological conditions, TSH via TSHR stimulates growth, differentiation and function of the thyroid cells [4,44]. This receptor is a target for THSRAb in Graves’ disease. It should be stressed that TSHR expression is common in breast cancer, with higher prevalence in low-grade breast cancer [13]. The clinical significance of the serum TSHRAb in relation to breast cancer and Graves' disease was unresolved in the previous observations [6,15]. In our study, TSHRAb levels were significantly higher in breast cancer comparing to controls. They were positive determinants of breast cancer in the univariate logistic regression analysis, which did not reach statistical significance in multivariate logistic regression analysis. However, in the analysis of the age-matched groups of patients, TSHRAb was found to be the only positive determinant of breast cancer. Therefore, we suggest that TSHRAb can be called a positive predictor for the subsequent development of breast cancer. However, further prospective research is required on a larger group of patients to determine unquestionably the significance of the above association.

Consequently, neutral TSHR antagonists (ligands inhibiting receptor activation by agonists), such as NIDDK-CEB-52, NCGC00242595 and NCGC00229600, could play a potential role in the breast cancer prophylaxis, acting as precursors of drugs preventing from TSHRAb activation in patients with Graves’ disease [45]. Monoclonal antibodies, currently used in experimental studies on the medical therapeutic intervention in Graves' disease by vaccination with chemically altered autoantigens, could selectively deplete specific T lymphocytes subsets and block the T-cell receptor MHC interaction [46]. Additionally, no reports were found assessing the theoretical suppressive effect of blocking subtype of TSHRAb (causing hypothyroidism) on breast cancer and thus such clinical research would be advisable.

As discussed above, reduced iodide concentration, elevated levels of the thyroid hormones and antibodies contributed to an increased risk of breast cancer in previous reports and in our study. However, some authors suggested also the possible impact of breast cancer on the thyroid with the resulting increased level of thyroid hormones and the autoimmune response with the detectable thyroid antibodies [5]. In our series we found shift of about 15–20 years between the primary peak of the diagnosed Graves' disease and the secondary maximal incidence of breast cancer (Figure 4). However, the observed long shift is partially affected by the fact that participants in our study were younger than the general population of patients with Graves’ disease, as they were hospitalized due to the more prominent symptoms. Older patients with usually more subtle clinical course of Graves’ disease are treated as outpatients [47]. Despite some possible level of bias, we claim that it is the Graves’ disease which could contribute to the subsequently observed development of breast cancer.

In conclusion, the strong relationship between Graves’ disease and breast cancer is proposed. We suggest that TSHRAb could be described as a positive determinant of breast cancer. The present data call attention to the usefulness of screening for breast cancer in long-term follow-up of patients with autoimmune thyroid disorders, especially of those with Graves’ disease. Similarly, screening for autoimmune thyroid disorders should be performed in patients with nodular breast disease. Additionally, the article draws ideas for further research in order to develop targeted treatment for more successful outcome in patients with breast cancer.

## Competing interests

The authors declare that they have no competing interests.

## Authors’ contributions

PS, WS, AL & MKL have made substantial contributions to conception and design of the study. PS and WS were involved in the acquisition of data. PS designed the data analysis program. All authors (PS, WS, AG, AL & MKL) performed analysis and interpretation of data. PS was involved in drafting the manuscript. MKL revised the draft critically for important intellectual content. AL & MKL supervised and guided all the steps of preparing the manuscript and gave final approval of the version to be published. All authors read and approved the final manuscript.
